# Procalcitonin and C-reactive protein levels at admission as predictors of duration of acute brain dysfunction in critically ill patients

**DOI:** 10.1186/cc10070

**Published:** 2011-03-02

**Authors:** Stuart McGrane, Timothy D Girard, Jennifer L Thompson, Ayumi K Shintani, Alison Woodworth, E Wesley Ely, Pratik P Pandharipande

**Affiliations:** 1 Department of Anesthesiology, Division of Critical Care, Vanderbilt University School of Medicine, 526 MAB, 1211 21st Ave South, Nashville, TN 37212, USA; 2 Department of Medicine, Division of Allergy, Pulmonary, and Critical Care Medicine and the Center for Health Services Research, Vanderbilt University School of Medicine, 6100 MCE, 1215 21st Ave South, Nashville, TN 37212, USA; 3 Geriatric Research, Education and Clinical Center (GRECC) Service, Department of Veterans Affairs Medical Center, 1310 24th Ave S, Nashville, TN 37212, USA; 4 Department of Biostatistics, Vanderbilt University School of Medicine, S2323 MCN, 1161 21st Ave South, Nashville, TN 37212, USA; 5 Department of Pathology, Division of Laboratory Medicine, Vanderbilt University School of Medicine, 4918 EA TVC, 1161 21st Ave. S, Nashville, TN 37232, USA; 6 Anesthesiology Service, Department of Veterans Affairs Medical Center, 1310 24th Ave S, Nashville, TN 37212, USA

## Abstract

**Introduction:**

Non-intensive care unit (ICU) cohorts have shown an association between inflammatory disturbances and delirium, though these relationships have not been studied in critically ill patients. This study sought to investigate the relationship between two inflammatory biomarkers, procalcitonin and C-reactive protein (CRP), and duration of acute brain dysfunction in ventilated patients.

**Methods:**

Patients enrolled in the Maximizing Efficacy of Targeted Sedation and Reducing Neurological Dysfunction (MENDS) trial were assessed daily for delirium using the Confusion Assessment Method-ICU. Plasma levels of procalcitonin and CRP were obtained within 24 hours of enrollment. Proportional odds logistic regression was used to examine the association between procalcitonin and CRP separately with delirium/coma-free days, adjusting for age, acute physiology score (APS) of the Acute Physiology And Chronic Health Evaluation (APACHE) II, sedation group (dexmedetomidine vs. lorazepam), and sepsis. Secondary analyses examined the association of these markers with other organ dysfunctions and 28-day survival.

**Results:**

Eighty-seven patients were included in this analysis. The median age of the patients was 60 years with APACHE II scores of 28; 68% had sepsis within 48 hours of admission. Higher levels of procalcitonin were associated with fewer delirium/coma-free days [odds ratio (OR), 0.5; 95% confidence interval (CI), 0.3 to 1.0; *P *= 0.04], whereas higher CRP levels showed trends towards fewer delirium/coma-free days (OR, 0.6; 95% CI, 0.3 to 1.1; *P *= 0.08). Similar relationships were found regardless of the presence of sepsis. No associations were found between procalcitonin or CRP with 28-day survival (*P *= 0.40 and 0.16, respectively).

**Conclusions:**

In our pilot study, high baseline inflammatory biomarkers predicted prolonged periods of acute brain dysfunction, implicating inflammation as an important mechanism in the pathophysiology of delirium and coma during critical illness, irrespective of whether patients had sepsis or not.

## Introduction

Delirium, a form of acute brain dysfunction, is a leading cause of morbidity and mortality in critically ill patients [1-6]. Recent studies in this important area of critical care have focused on elucidating risk factors of delirium and developing strategies to reduce the prevalence and duration of delirium, but little work has been done to determine the mechanisms causing delirium in critically ill patients [3,7-14].

Present theories regarding the pathogenesis of delirium postulate a role for neurotransmitter imbalances (dopamine, norepinephrine, acetylcholine and serotonin), amino acids perturbations, oxidative stress, and inflammation [15-18]. Studies have shown that higher levels of C-reactive protein (CRP) and interleukin (IL)-6 are associated with a greater incidence of delirium in postoperative hip surgery patients [19-21]. These biomarkers may be general measures of complex inflammatory processes that promote delirium, or they may directly contribute to brain dysfunction. CRP, for example, can incite the formation of reactive oxygen species, causing disruption of the blood brain barrier and resultant neuronal dysfunction, which may manifest as delirium [19-21]. Procalcitonin has recently gained popularity as an early marker for sepsis [22,23], though no study to our knowledge has examined the role of this or other inflammatory biomarkers in delirium in critically ill mechanically ventilated patients.

The primary aim of our prospective cohort study was to test the hypothesis that systemic inflammation, as measured by the inflammatory biomarkers procalcitonin and CRP, is associated with a longer duration of brain dysfunction in mechanically ventilated patients.

## Materials and methods

This prospective cohort study was nested within the Maximizing Efficacy of Targeted Sedation and Reducing Neurological Dysfunction (MENDS) [13] double-blind, randomized controlled trial (Trial Registration: clinicaltrials.gov identifier: NCT00095251). This trial compared dexmedetomidine and lorazepam for sedation during acute mechanical ventilation, with both groups receiving fentanyl for analgesia. The patient population has previously been described in detail: adult mechanically ventilated, medical and surgical ICU patients from two tertiary care centers were enrolled between August 2004 and April 2006, after excluding those in whom delirium could not be reliably identified (for example, due to previous stroke, cerebral palsy, severe dementia, severe hearing disabilities, or inability to understand English), patients with active seizure disorder, Child-Pugh class B or C cirrhosis, alcohol abuse, active myocardial ischemia, second- or third-degree heart block, and pregnancy [13]. The institutional review board (IRB) at Vanderbilt University, Nashville, Tennessee, approved this study and informed consent was obtained from patients or their authorized surrogates. Patients for whom surrogate consent was obtained were re-consented once competent to provide informed consent.

At enrollment, we collected demographics and clinical characteristics from the computerized medical record and determined previous cognitive function through surrogate interview using the validated Informant Questionnaire on Cognitive Decline in the Elderly (IQCODE) [24].

Blood samples for measurement of procalcitonin and CRP were collected within 24 hours of enrollment and centrifuged. Plasma was separated and stored at -80°C. Procalcitonin was measured by Time Resolved Amplified Cryptate Emission (TRACE) Assay analysis on the B.R.A.H.M.S. Kryptor^® ^Compact instrument, and CRP was analyzed by the Roche Cobas Integra 800 analyzer (Roche Diagnostic, Indianapolis, IN, USA). Both procalcitonin and CRP have previously been shown to be stable at room temperature and 4°C [25,26]. Additionally, blood sampling technique (for example, arterial vs. venous line) and repeated freezing/thawing cycles have no significant influence on the procalcitonin or CRP concentrations [25].

Delirium was assessed daily until hospital discharge or for up to 12 days using the Confusion Assessment Method for the Intensive Care Unit (CAM-ICU) [27,28]. Level of consciousness was measured with the Richmond Agitation-Sedation Scale (RASS) [29,30]. Patients were considered delirious if their RASS score was -3 or greater (that is, -3 to +4) and they had a positive CAM-ICU per the original validation studies of the CAM-ICU [27,28]. Coma was defined as a RASS score of -4 (responsive to physical but not to verbal stimulus) or -5 (unresponsive to verbal and physical stimulus) [27,28]. Patients who were comatose were unable to be evaluated for delirium, since delirium evaluation with the CAM-ICU requires a patient to be responsive to voice.

For the purpose of this study, we chose delirium/coma-free days (DCFD) as our outcome measure to indicate the days during the 12-day study period that a patient was alive and free of acute brain dysfunction (delirium and coma); thus, this outcome reflects days that a person has "normal" mental status as evaluated by the CAM-ICU [13]. This outcome was chosen over delirium days to avoid confounding by death, which cuts short the duration of brain dysfunction if a patient dies early, artificially improving neurologic outcomes. Furthermore, delirium-free days (DFDs) was not chosen as an outcome, since in DFDs even patients who are comatose (as against normal) are considered "delirium-free;" thus DFDs is not representative of better neurological outcomes in populations where coma is common [13]. Since DCFDs reflects not only brain dysfunction but is also influenced by survival, we additionally assessed the associations of procalcitonin and CRP with other organ dysfunction-free days (for example, kidney-, lung-, liver-, coagulation- and hemodynamic-dysfunction free days) and 28-day survival. If the biomarkers are associated with DCFDs primarily because of a relationship with survival (rather than a brain-specific relationship), then all organ dysfunction-free days should show a similar association. The organ dysfunction definitions were adapted from the Sequential Organ Failure Score (SOFA) score cut-offs: kidney, Cr >2 mg/dL or urine <400 cc/day; lung, PaO_2_/FiO_2 _<300 or SaO_2_/FiO_2 _<315 [31]; liver, total bilirubin >2 mg/dL; coagulation, platelet count <100,000/mm^3^; and hemodynamic, need for vasopressor [32,33], consistent with definitions utilized in published studies of organ dysfunction in critically ill patients [34].

### Statistical analysis

Baseline demographic and clinical variables are presented using medians and interquartile ranges for continuous variables and proportions for categorical variables. Because the distribution of DCFDs was heavily skewed, we used proportional odds logistic regression to determine the relationship between each biomarker (baseline plasma procalcitonin or CRP, separately) and our primary outcome of DCFDs, after adjustment for age, APACHE II acute physiology score at enrollment, treatment group (dexmedetomidine or lorazepam), and sepsis. In our multivariable model, we wanted to adjust for physiological changes that would confound the relationship between the inflammatory biomarkers and delirium and also independently assess the role of age in delirium. Since age is already incorporated into the APACHE scoring system, this would have led to age being accounted for twice in our models if we chose the APACHE II as a covariate. Thus using the physiological score from the APACHE served as a better covariate in our model. In addition to the proportional odds model, point estimates and 95% confidence intervals were calculated using bootstrapped linear regression to estimate the difference in number of DCFDs between patients with values of procalcitonin or CRP at the 25^th ^vs. 75^th ^percentiles of our population. To analyze the relationships between the biomarkers and our secondary outcomes of other organ dysfunction-free days and 28-day survival, we used proportional odds logistic regression and Cox proportional hazards regression, respectively, adjusting for the same covariates. For Cox regression, patients were censored at the time of last contact or at study Day 28, whichever came first. Due to its skewed distribution, procalcitonin concentrations were log transformed prior to all analyses in order to provide better model fits. Given that CRP and (in a more specific way) procalcitonin are increased in sepsis, we evaluated the hypothesis that the associations between these biomarkers and outcomes could be influenced by the presence or absence of sepsis by including an interaction term (inflammatory biomarker x sepsis) in each regression model. In all regression models, we also evaluated the associations between biomarker concentrations and outcomes for nonlinearity by including restricted cubic splines. Nonlinear terms and interaction terms were removed from each regression model if there was no evidence of nonlinearity or an interaction, respectively (that is, if *P *for nonlinearity or interaction >0.20). All reported odds ratios (ORs) and 95% confidence intervals (CIs) estimate the odds of more vs. fewer dysfunction-free days among patients at the 75^th ^percentile of the biomarker levels of our population vs. the 25^th ^percentile (a more clinically relevant distinction than the traditional one-unit change in biomarker). We used R version 2.9.1 for all statistical analyses (R Development Core Team (2009); Vienna, Austria).

## Results

Of the 103 patients enrolled in the MENDS study [13], baseline procalcitonin and CRP data were available for 87 subjects who were included in the final analysis (16 patients had inadequate plasma samples for CRP and procalcitonin measurements). Baseline characteristics of the study population are listed in Table 1. The median APACHE II and SOFA scores were high at baseline, indicating this group of patients was critically ill; 68% of the population was diagnosed with sepsis within 48 hours of admission to the ICU using the standard definition of suspected infection accompanied by at least two SIRS (systemic inflammatory response syndrome) criteria.

**Table 1:** Baseline demographics and clinical characteristics

Variable*	*N *= 88
Age (years)	60 (49, 66)
Males	50%
APACHE II	28 (24, 32)
SOFA score	9 (8, 12)
Sepsis	68%
Brain dysfunction at enrollment	
Coma	53%
Delirium	30%
Baseline RASS score	
-4 or -5	52%
-3 or -2	27%
-1 to +1	21%
Baseline procalcitonin level (ng/ml)	1.53 (0.43, 6.74)
Baseline CRP level (ng/ml)	196 (107, 282)
Ever on vasoactive agents	55%
Duration of vasoactive agent use	1 (0, 3)
Days on mechanical ventilation	5.6 (3.2, 12.3)
ICU length of stay	9.2 (5.2, 17.4)
Hospital length of stay	15.6 (7.9, 23.5)
28-day mortality	26%

*Median (interquartile range) unless specified.

Abbreviations: APACHE II, Acute Physiological and Chronic Health Evaluation II; CRP, C-reactive protein; RASS, Richmond Agitation-Sedation Scale; SOFA, Sequential Organ Failure Score.

### Associations of biomarkers with delirium/coma-free days

**Figure 1:**
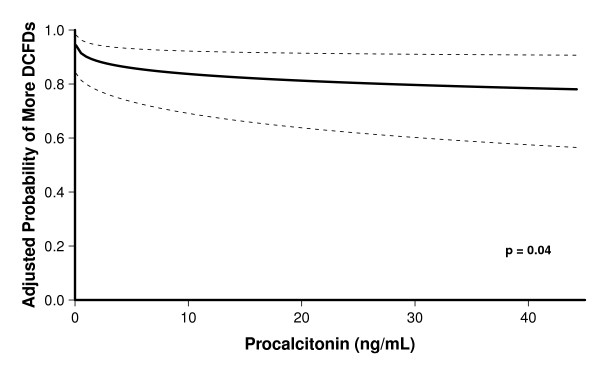
**Association between baseline procalcitonin and delirium/coma-free days**. The dark line represents the point estimates for the probability of the outcomes, while the dotted lines represent the confidence intervals. The odds of having more delirium/coma-free days (DCFDs) was significantly reduced with increasing levels of procalcitonin (OR, 0.5; 95% CI, 0.3 to 1.0; *P *= 0.04). Thus, a patient with a baseline procalcitonin value of 6.7 ng/ml (the 75th percentile value) would have half the odds of having more DCFDs, as a patient with a baseline procalcitonin of 0.4 ng/ml (the 25th percentile value).

**Figure 2:**
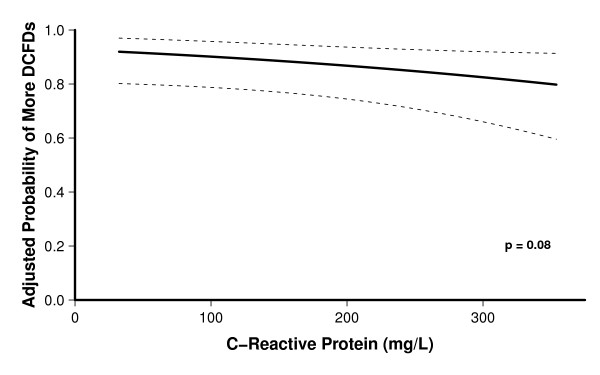
**Association between baseline C-reactive protein (CRP) and delirium/coma-free days**. The dark line represents the point estimates for the probability of the outcomes, while the dotted lines represent the confidence intervals. The odds of having more delirium/coma-free days (DCFDs) was reduced with increasing levels of CRP (OR, 0.6; 95% CI, 0.4 to 1.1; *P *= 0.08). Thus, a patient with a baseline CRP value of 281.5 mg/L (the 75th percentile value) would have 0.6 times the odds of more DCFDs as a patient with a baseline CRP of 107 mg/L (the 75th percentile value).

After adjusting for covariates, higher baseline procalcitonin levels were associated with fewer DCFDs, that is, more brain dysfunction (OR, 0.5; 95% CI, 0.3 to 1.0; *P *= 0.04; Table 2 and Figure 1), and higher baseline CRP levels showed a trend towards fewer DCFDs (OR, 0.6; 95% CI, 0.3 to 1.1; *P *= 0.08; Table 2 and Figure 2). Patients with a baseline procalcitonin level of 0.4 ng/ml (the 25^th ^percentile value in our study population) had a mean (95% CI) of 1.2 (0.1, 2.5) more days alive and free of delirium and coma than those with a procalcitonin level of 6.7 ng/ml (the 75^th ^percentile). Similarly, patients with a baseline CRP level of 107 mg/L (the 25^th ^percentile) had a mean (95% CI) of 1.0 (-0.25 to 2.3) more day alive and free of delirium and coma than those with a CRP level of 281.5 mg/L (the 75^th ^percentile). Sepsis did not modify the relationships between the biomarkers and DCFDs (that is, no interactions were present); thus, patients with higher levels of procalcitonin or CRP had fewer delirium/coma-free days irrespective of whether they had sepsis or not.

**Table 2:** Inflammatory biomarkers and outcomes

	Inflammatory Biomarker Odds Ratio (95% Confidence Intervals)*‡	
	
Outcome	Procalcitonin	C-reactive protein
**Delirium/coma-free days**	0.5 (0.3, 1.0)	0.6 (0.3, 1.1)
**28-day survival**^**†**^	1.4 (0.7, 2.9)	1.7 (0.8, 3.4)
**Kidney-dysfunction free days**	0.7 (0.3, 1.3)	---
Septic	---	0.3 (0.1, 0.6)
Non-Septic	---	2.6 (0.5, 12.4)
**Lung-dysfunction free days**	1.0 (0.5, 1.9)	0.4 (0.2, 0.8)
**Liver-dysfunction free days**	1.1 (0.5, 2.3)	0.9 (0.4, 1.9)
**Coagulation-dysfunction free days**	1.0 (0.5, 1.9)	
Septic	---	1.1 (0.4, 2.6)
Non-Septic	---	2.1 (0.3, 14.9)
**Hemodynamic-dysfunction free days**	0.4 (0.2, 0.8)	---
Septic	---	0.6 (0.2, 1.4)
Non-Septic	---	12.4 (0.7, 229.8)
**Cardiac-dysfunction free days**	0.6 (0.3, 1.3)	
Septic	---	0.7 (0.3, 1.5)
Non-Septic	---	3.0 (0.5, 16.6)

*All reported ratios compare the odds or hazard of the outcome (for example, more dysfunction-free days) among patients at the 75^th ^percentile of the biomarker levels of our population to patients at the 25^th ^percentile. This is a more clinically relevant comparison than a one-unit change in biomarker and allows for some description of nonlinear association.

‡We assessed for interactions between the presence of sepsis and the association of the biomarkers with each organ dysfunction outcome. A single odds ratio is presented when sepsis did not affect the association of the biomarker with the outcome. If a significant interaction with sepsis existed, however, then two separate odds ratios are presented to show the different associations between the biomarker and the organ dysfunction, depending on the presence of sepsis.

†Hazard ratios are presented for 28-day survival.

### Associations of biomarkers with survival and other organ dysfunction-free days

Baseline procalcitonin was not associated with survival (*P *= 0.40), nor was it associated with duration of lung dysfunction (*P *= 0.95), liver dysfunction (*P *= 0.87), coagulation dysfunction (*P *= 0.98) or kidney dysfunction (0.23) (Table 2), suggesting that the association between procalcitonin and DCFDs is a brain-specific association rather than a result of an association with reduced survival. High baseline procalcitonin levels were associated with fewer days free of hemodynamic dysfunction (*P *= 0.007); no interaction with sepsis was found (that is, this association was present whether the patient had sepsis or not) (Table 2).

CRP was not associated with survival (*P *= 0.16), duration of liver dysfunction (*P *= 0.74), coagulation dysfunction (*P *= 0.40), or hemodynamic dysfunction (*P *= 0.16). Greater baseline CRP level was associated with lower probability of kidney and lung dysfunction-free days (*P *= 0.005 and 0.008, respectively). The relationship between CRP and kidney-dysfunction free days was significantly modified by sepsis (interaction *P *= 0.01) (Table 2). Thus, among the septic patients, a significant association existed between CRP and kidney-dysfunction free days, such that the highest CRP concentrations were associated with a lower probability of kidney-dysfunction free days, after adjusting for covariates. However, among non-septic patients, this association was not significant.

## Discussion

To our knowledge this is the first study to show that inflammation, as measured by procalcitonin and CRP, is independently associated with acute brain dysfunction in critically ill, mechanically ventilated patients. Specifically, we found that high concentrations of procalcitonin were associated with fewer days alive and free of delirium and coma (meaning fewer days alive and free of brain dysfunction), and CRP showed a trend toward a similar relationship, after adjusting for potential confounders. No consistent association was found between these biomarkers and other organ dysfunctions (that is, some were related but most, along with survival, were not), indicating the associations with duration of brain dysfunction are likely organ-specific.

The findings of our study suggest inflammation may be one important contributor to acute brain dysfunction (delirium and coma) in ICU patients, irrespective of the presence or absence of sepsis or the influence of inflammation on survival and other organ dysfunctions [35-38]. Inflammatory mediators produced during critical illness (for example, tumor necrosis factor-α, interleukin-1) initiate a systemic cascade of endothelial damage, thrombin formation, and microvascular compromise [39]. Studies in animal models have revealed that these inflammatory mediators cross the blood-brain barrier [40], increase vascular permeability in the brain [41], and result in changes on electroencephalography (EEG) that are consistent with those seen in patients with delirium [42,43]. Inflammation may also incite brain dysfunction by constricting cerebral vasculature through activation of α_1_-adrenoceptors [44] or by interfering with neurotransmitter synthesis and neurotransmission [15].

There is debate within the literature whether procalcitonin is a specific marker of infection, though receiver operating curve (ROC) characteristics in some studies suggest that the performance of procalcitonin as a marker of sepsis is superior to that of CRP [22,23]. CRP on the other hand, is thought to be a general marker of systemic inflammation, of which infection is one common cause [45]. As we found an association between procalcitonin levels and DCFDs in patients with sepsis and in those without sepsis, our results suggest that inflammation due to sepsis was not the sole driver of procalcitonin's association with DCFDs. Additionally the trend shown with CRP supports our hypothesis that inflammation of either infectious or non-infectious etiologies is associated with ICU delirium and coma. Larger cohort studies are needed to elucidate the contribution of specific causes of inflammation to acute brain dysfunction in the ICU.

There are several strengths and limitations in our study. First, this pilot investigation, which evaluated 87 patients, may lack adequate statistical power to detect some clinically important associations. Furthermore, the limited number of patients prevented us from studying potential differences in various subgroups such as surgical versus medical ICU patients; for example, some data suggest that post-surgical patients have elevated levels of procalcitonin dependent on the nature and extent of surgery [46,47]. Despite these limitations, we were able to show associations between procalcitonin and CRP with DCFDs, providing a basis for studying the role of inflammation in delirium and coma in the ICU. Second, because we studied only baseline procalcitonin and CRP levels, we were unable to assess whether changes in these inflammatory markers over time are associated with the resolution or persistence of brain dysfunction. Third, because of our small sample size, only a limited number of covariates could be included in our regression models; the inclusion of additional potential confounders would have increased the possibility of obtaining unreliable results due to overfitting. We did, however, adjust for sepsis, which represents a major risk factor. Furthermore, our results are consistent with studies that have shown an association of CRP with delirium in non-ICU cohorts [19-21], delirium following stroke [48] and sepsis [49], and delirium in one small study of 32 ICU patients, which did not adjust for confounders [50]. Additionally, we found that among septic patients, higher baseline CRP levels were associated with a greater duration of renal dysfunction, which is in concordance with a previous study by Lobo *et al. *[51] that showed that increased admission CRP was proportional to days of renal support therapy. Fourth, our chosen outcome of delirium/coma-free days (representing the days alive without delirium or coma) can be influenced by death, which in turn may be dependent on other organ dysfunctions. We assessed the role of both procalcitonin and CRP in non-brain organ dysfunctions and survival and found little evidence that the observed associations between the biomarkers and delirium/coma-free days were driven by a relationship with survival. In fact, there were no associations between the biomarkers and survival in our study. Finally, we assessed patients only once a day for delirium, and patients who were CAM-ICU negative were considered to be "free of brain dysfunction" for the entire day. Given that delirium is a fluctuating state of mental status, it is possible that some patients developed delirium later in the day and were missed in our study.

## Conclusions

In this pilot study, inflammation, as measured by high procalcitonin and CRP levels at admission, was associated with fewer days alive and free of acute brain dysfunction in critically ill patients, suggesting that inflammation plays an important role in delirium and coma in the ICU. Future studies are needed to elucidate further the inflammatory mechanisms of delirium and coma during critical illness.

## Key messages

• Delirium is a highly prevalent organ dysfunction in the critically ill with associated morbidity and mortality.

• Present theories regarding the pathogenesis of delirium postulate a role for neurotransmitter imbalances (dopamine, norepinephrine, acetylcholine and serotonin), amino acids perturbations, oxidative stress, and inflammation.

• In this prospective cohort study of critically ill patients, increasing levels of procalcitonin at admission were associated with longer duration of brain dysfunction. C-reactive protein showed similar trends.

• The association between increasing biomarkers and worse brain dysfunction was independent of sepsis; thus inflammatory perturbations of either infective or non-infective etiology were both associated with delirium.

• There was not a consistent association between procalcitonin or C-reactive protein and other organ dysfunctions or death implying that inflammation may have a bigger impact on the brain than just global organ dysfunction.

## Abbreviations

APACHE: Acute Physiology and Chronic Health Evaluation; APS: acute physiology score; CAM-ICU: Confusion Assessment Method for the Intensive Care Unit; CI: confidence interval; CRP: C-reactive protein; DCFD: delirium/coma-free days; DFD: delirium-free days; EEG: electroencephalography; ICU: intensive care unit; IL: interleukin; IQCODE: Informant Questionnaire on Cognitive Decline in the Elderly; IRB: institutional review board; MENDS trial: Maximizing Efficacy of Targeted Sedation and Reducing Neurological Dysfunction trial; OR: odds ratio; RASS: Richmond Agitation-Sedation Scale; ROC: receiver operating curve; SOFA: Sequential Organ Failure Score.

## Competing interests

Drs. Girard, Pandharipande, Shintani, and Ely have received honoraria from Hospira Inc. Drs. Pandharipande and Ely have received grant support from Hospira Inc. Dr. Ely has also received grant support from Pfizer Inc., Eli Lilly and Co., Glaxo-Smith-Kline, and Aspect Medical Systems and is an advisor to Healthways Inc. All other authors declare they have no competing interests.

## Authors' contributions

SM contributed to the study design, acquisition of data, analysis and interpretation of results, drafting of the manuscript and critical revisions of the manuscript for intellectual content. TG contributed to the study design, acquisition of data, analysis and interpretation of results, and critical revisions of manuscript for intellectual content. JT and AS contributed to the study design, statistical analysis and interpretation of results, and critical revisions of manuscript for intellectual content. AW contributed to development of methodology and analysis of procalcitonin and C-reactive protein, analysis of the results, and critical revisions of manuscript for intellectual content. EWE contributed to the study design, acquisition of data, analysis and interpretation of results, and critical revisions of manuscript for intellectual content. PP contributed to the study design, acquisition of data, analysis and interpretation of results, drafting of manuscript and critical revisions for intellectual content. All authors read and approved the final manuscript.
